# Malocclusion in Elementary School Children in Beirut: Severity and Related Social/Behavioral Factors

**DOI:** 10.1155/2015/351231

**Published:** 2015-01-26

**Authors:** Antoine Hanna, Monique Chaaya, Celine Moukarzel, Khalil El Asmar, Miran Jaffa, Joseph G. Ghafari

**Affiliations:** ^1^Division of Orthodontics and Dentofacial Orthopedics, Department of Otolaryngology/Head and Neck Surgery, American University of Beirut Medical Center, Beirut, Lebanon; ^2^Department of Epidemiology and Population Health, Faculty of Health Sciences, American University of Beirut, Beirut, Lebanon; ^3^Private Practice, Beirut, Lebanon

## Abstract

*Aim*. To assess severity of malocclusion in Lebanese elementary school children and the relationship between components of malocclusion and sociodemographic and behavioral factors. *Methods*. Dental screening was performed on 655 school children aged 6–11 from 2 public (PB) and 5 private (PV) schools in Beirut. A calibrated examiner recorded occlusion, overjet, overbite, posterior crossbite, midline diastema, and crowding. Another examiner determined the DMFT (Decayed/Missing/Filled Teeth) score. A questionnaire filled by the parents provided data on sociodemographic and behavioral factors. Multinomial, binomial, and multiple linear regressions tested the association of these factors with occlusal indices. *Results*. Malocclusion was more severe in PB students. Age and sucking habit were associated with various components of malocclusion. Crowding was more prevalent among males and significantly associated with the DMFT score. Income and educational level were significantly higher (*P* < 0.05) in PV pupils and deleterious habits were more frequent in PB children. *Conclusions*. Children of lower socioeconomic background had more severe malocclusions and poorer general dental health. Compared to Western and WHO norms, the findings prompt health policy suggestions to improve dental care of particularly public school children through regular screenings in schools, prevention methods when applicable, and cost effective practices through public and private enabling agencies.

## 1. Introduction

Malocclusion is defined as any deviation from the norm of the arrangement of the teeth and occurs commonly among various populations [1, 2]. While considered nonlife threatening, malocclusion may cause altered functions (mastication, speech) and poor dentofacial esthetics that reduce the quality of life of affected subjects including social and functional limitations [3]. Malocclusion has also been associated with the development of periodontal disease, albeit not a direct etiology [4].

The assessment of malocclusion has not been uniform. Relatively subjective weights are assigned to the components of malocclusions in different rating systems [5], eventually leading to variable reporting of orthodontic treatment need. The corresponding scoring indices have been used by governmental and insurance agencies to determine eligibility and/or amount of treatment coverage.

Prevalence of malocclusion in the deciduous (primary), intermediate, and permanent dentitions varied widely across studies and countries because of population differences (races/ethnicity), sample sizes, age range of the surveyed children, and methods of measurement [6–10]. Yet, fewer differences were found in classification of malocclusion because of more standardized norms of the relationship between maxillary and mandibular molars (molar occlusion) or the overjet (horizontal overbite) between upper and lower incisors. In general, these relations are well correlated [11].

In Western Studies (mainly American) spanning over 50 years, the majority of malocclusions in Caucasian children exhibit Class I malocclusion (nearly 75%), with closer to normal relations between the posterior teeth, followed by Class II malocclusion (tendency to increased overjet, nearly 20%), then Class III (anterior underbite-less than 5%) [2]. Surveys of Brazilian children indicated lower proportions but still a majority of Class I malocclusion and higher percentages of Class II and Class III problems [12, 13].

Fewer studies are available on prevalence of malocclusion in the Middle East and Northern Africa region, but the same pattern of malocclusion as in Western countries seemingly holds, although in varied proportions. The majority of malocclusions in Middle Eastern countries related to Class I in children, adolescents, or young adults, followed by Classes II and III [14–18]. In some studies the components of the malocclusion were further qualified [14, 16–18].

Although scarce, studies that have directly related malocclusion and its severity to social status indicated that children with relatively poor lifestyle have higher orthodontic treatment need compared to their counterparts with wealthier lifestyle [19, 20]. Social condition also can affect malocclusion indirectly. Underprivileged people are more exposed to risk factors that affect oral health: unhealthy diet, tobacco use, excessive consumption of alcohol, poor sanitation and polluted water, poor oral hygiene, and HIV infection [21]. Poor oral health leads to caries and early tooth loss, facilitating the development of malocclusion, hence greater need for orthodontic treatment [22, 23].

The available data on malocclusion in Lebanon only cover the age range of 9–15 years. Prevention of oral disease or dysmorphology is usually implemented at an earlier age, thus the importance of assessing the prevalence of malocclusion at younger ages (6–11 years). Relating malocclusion to social/behavioral factors in early childhood shall facilitate the prevention or decrease in severity of malocclusion, particularly in the presence of guidelines recommending intervention before the emergence of all permanent teeth (usually by age 12 years) [24].

Accordingly, our aim was to evaluate in prepubertal Lebanese students from presumably varied backgrounds, attending public and private schools, the prevalence of malocclusion and its relation to social and behavioral characteristics. Such information potentially helps public health workers to plan intervention programs and highlight the importance of early orthodontic screening.

## 2. Material and Methods

The investigation was a comparative cross-sectional study of elementary school children in grades 2 to 5, aged 6–11 years, attending public and private schools in Beirut, Lebanon. The data were collected through an oral examination and a questionnaire sent to parents or guardians. The Institutional Review Board of the American University of Beirut and the Ethics committees of all participating institutions approved the study along with the pertinent consent forms secured by the parents and the assent provided by children.

### 2.1. Sample Size Calculation

A sample size of 721 was determined through the* A Priori Sample Size Calculator for Multiple Regression* [25] with an anticipated effect size (f2) of 0.02, a statistical power level of 0.8, 7 predictors, and a probability level of 0.05. Accounting for probable missing data we inflated the sample size by 1.2; for nonresponse from the parents or the children we further inflated the size by 1.25, whereby we sought to approach a number of nearly 1000 children.

### 2.2. Participants

Access to public schools was possible through a local nongovernmental organization (NGO, “Ajialouna”) in Beirut, dedicated to improve life standards through various projects such as school health, health education programs, orphan sponsorship and other commitments. From a total of 30 public schools, two schools were chosen based on a timetable provided by the NGO indicating the readiness of schools for the survey and prior consent from parents that cleared our conduct of oral examination on 530 children. However, only 325 (61.3%) of these students were recruited, because the parents of the remaining 205 children did not consent to participate in the survey. The findings of the oral examinations of these pupils were used only to determine potential differences with the consenting participants.

Private schools were selected based on location (Beirut and suburbs) and willingness to participate. From 12 contacted schools, five agreed to partake in the study, encompassing 1119 children from average to high socioeconomic status. The parents/guardians of 333 children (29.76%) agreed to enroll their child and answered the survey. Excluding 3 subjects with prior or current interceptive orthodontic treatment, the final number was 330. The comparatively low response rate was probably related to the prior dental screening at the start of the school year in some of the schools, or to a more regular follow-up by a private dentist. The total sample size of both private and public schools was 655.

### 2.3. Instruments

The components of the US National Health and Nutrition Estimates Survey (NHANES) malocclusion assessment model were used. They included spacing within the arch (crowding, midline diastema) and relations between maxillary and mandibular teeth in the 3 planes of space: sagittal (relations between anterior teeth: overjet or anterior crossbite; relationship between the first permanent molars (Class I, II, or III)), vertical (overbite or open bite), and transversal (posterior crossbite). The study deviated from the NHANES gauge in 2 aspects.

1. A more complete description of the malocclusion was added by dividing the molar occlusion into 5 categories based on half-cusp deviation and recording the overbite not only in millimeters but also as percentage of overlap of the mandibular incisors by the maxillary incisors.
2. The maxillary irregularity index was discarded because the sample age bracket (6–11 years) was lower than the NHANES range (8–11 years), precluding the examination of a large number of children with nonerupted maxillary lateral incisors. Given a high correlation between the maxillary and mandibular irregularity scores [2], the latter increasing more from childhood to adulthood, we projected the mandibular score to properly represent the presence and severity of crowding. Additional findings worth reporting were noted separately (missing and supernumerary teeth, impeded eruption of teeth).

Outcome was classified into quantitative measurements (number of teeth in crossbite and percentage of overbite), nominal measurements (molar and canine occlusion), or an ordinal variable reflecting severity (crowding, overjet, and overbite).

The DMFT (Decayed/Missing/Filled Teeth) score and the plaque index (a measure of hygiene) were recorded for each child to be analyzed in a different paper.

The questionnaire addressed to parents included 41 questions in the following categories:

- sociodemographic background of child and parents (family status and educational background of the respondent, family monthly income, and child's birth order),
- general health status of the child (presence/absence of chronic disease, child's breathing mode, and smoking status of the mother during pregnancy),
- sucking habits of the child (digit or other object, duration, and intensity of sucking),
- feeding methods of the child (feeding mode of child during infancy, consumption of detrimental foods),
- oral health behaviors (brushing habits, visits to the dentist),
- perception of parents towards their child's oral health (malocclusion and decays).

### 2.4. Procedure

Calibration studies preceded the in-field examinations. The dental examination comprised two distinct parts: the collection of occlusal data (investigator AH); the determination of the DMFT and plaque scores (investigator CM). The dental instruments used were noninvasive mouth mirrors, probes, and periodontal probes (ZFA043#11, Co), available in disposable packages. The screenings were performed according to WHO standards [26].

A document was sent with the children to their parents, including all pertinent information regarding the study, the IRB-approved questionnaire, and consent form. When the child was found to require treatment, a note was sent to the parents or legal guardian(s). Essential information contacts of nearby specialized dental centers with reasonable treatment cost were provided to the parents when the child was not being followed up by a dentist.

### 2.5. Data Analysis

Frequency distributions for all variables helped assess variability and data regrouping. Outcome indicators were chosen to represent each plane of space: overjet, overbite, and posterior crossbite. Bivariate associations gauged how different malocclusion components vary relative to selected characteristics, through chi-square tests or independent sample *t*-tests between each dependent variable and the study covariates, depending on the nature of the variables.

Multivariate analysis was performed using the generalized estimated equations (GEEs), to estimate coefficients and odds ratios by fitting regression models with continuous (overjet in mm) and binary (presence/absence of posterior crossbite) outcomes adjusted for clustering effect. As GEE does not model multinomial outcome variables, generalized linear models (GLM) were used to estimate relative risk ratios (RRR) by fitting multinomial logistic regression models for outcome variables having more than 2 categories (overjet severity, overbite severity, and irregularity score severity). Clustering effect was adjusted for in the variance-covariance matrix structure and robust standard errors were reported. The multinomial regression was used instead of the ordinal logistic regression because the proportional odds assumption did not hold.

All covariates statistically associated with outcome variables at the bivariate level of *P* < 0.2 were included in the multivariate analysis. For all parameters, 95% CI and two-sided *P* values were reported. Statistical significance was set at *P* < 0.05. All analyses were completed in Stata SE 10.1.

## 3. Results

### 3.1. Sociobehavioral Characteristics

Mean age was not significantly different between private schools [PV] (8.57 ± 1.31 yrs.) and public schools [PB] (8.49 ± 1.59 yrs.) children. The proportion of girls was slightly higher in PV (52.8%) than in PB (46.2%) schools, but the difference was not statistically significant.

Statistically significant differences were found between both groups for family income and educational level of parents, the higher levels detected among parents of PVS children (Table 1). Regarding behavioral factors, the proportion of children whose mothers smoked during pregnancy was nearly 3 times higher among PB (20.4%) compared to PV schools. Reported sucking habits were also higher for PB children (Table 2).

### 3.2. Dental Measures

To facilitate the communication of a large set of data, only malocclusion parameters (by type of school) with statistically significant differences between PB and PV children are displayed in Table 3. The largest proportions of PB and PV children had Class I (normal) occlusion. The type of occlusion classified by molar relations (Class I, II, or III) was not statistically significantly different between school groups; however, when occlusion was stratified based on overjet severity the differences were significant (Table 3). PB children had statistically significantly greater mean and higher percentage of OJ compared with PV children. Anterior crossbite (reverse overjet) was statistically significantly different between the groups in Class III malocclusions. Midline diastema was more prevalent in public compared to private schools.

Vertical measures (open bite, overbite) were not statistically significantly different between school groups; however, transverse abnormalities (posterior crossbite and midline diastema) were more frequent in PB. The overall DMFT scored a mean of 7.30 ± 3.98 in PB children compared with a mean of 3.50 ± 3.41 in PV schools (*P* < 0.0001). The number of decayed teeth was significantly higher in PB compared to PV (*P* ≤ 0.0001) schools, with means of 5.67 ± 3.81 and 1.48 ± 2.19, respectively.

### 3.3. Associations among Variables

#### 3.3.1. Bivariate Associations

Only statistically significant associations are displayed in Table 4. Overjet was statistically significantly associated with age (6-7 years when permanent incisors and first molars erupt, and 8–11 years prior and during the eruption of permanent canines and premolars) and DMFT score. DMFT scores were significantly higher in children with more severe overjet. Overbite was significantly associated with age and plaque index. A higher proportion of older children had severe overbite compared to younger ones. The mean plaque index was greater in subjects with deeper bite. The post hoc test showed that the statistically significant difference existed between the subjects with moderate and deep bite (*P* = 0.043). Children with sucking habits were almost twice more likely to have at least one tooth in posterior crossbite compared to those with no sucking habit. None of the occlusal variables were associated with the amount or severity of the irregularity index.

#### 3.3.2. Multivariate Analysis

The clustering by school did not appear to have any effect on the regression outcome of the overjet, overbite, and irregularity index. However, for the posterior crossbite, age only became significant following adjustment for clustering.

Adjusting for gender, school type, educational level, sucking duration, DMFT score, and plaque index, the results indicate that a subject older than 8 years is at higher risk to have a mild rather than an ideal overjet (RRR: 1.35; 95% CI: 1.04–1.28; Table 5). Children with higher plaque index were at a lower risk of having a severe overjet (RRR: 0.93; 95% CI: 0.88–0.98).

When using the overjet as a continuous outcome and adjusting for the same covariates employed in the multinomial model, the regression model resulted in a positive correlation between age and overjet (*β*: 0.14; 95% CI: 0.046–0.249; Table 5). PV students were more likely to have a lower overjet than those attending public school (*β*: −0.10; 95% CI: −0.185; −0.026). Family income was positively associated with overjet, children of lower income families (<500,000 LL) exhibiting a greater likelihood for increased overjet.

Subjects 8–11 years of age were at a higher risk of having mild (RRR: 1.71; 95% CI: 1.21; 2.39) and moderate to severe (RRR: 2.23; 95% CI: 1.03; 4.83) overbite (Table 5). Also, children with increased sucking habit duration (RRR: 0.98; 95% CI: 0.97; 0.99) and higher DMFT score (RRR: 0.93; 95% CI: 0.86; 0.99) were at a lower risk of reporting moderate to severe overbite.

The odds of having posterior crossbite in 8–11-year old children and those with increased sucking habit duration were 1.29 (95% CI: 1.18; 1.39) and 1.01 (95% CI: 1.01; 1.18), respectively. Subjects with mouth breathing habit were more likely to have a mild irregularity index (RRR: 2.61; 95% CI: 1.99; 3.42). A higher risk of moderate to severe irregularity index was determined for male subjects (RRR: 1.69; 95% CI: 1.36; 2.1) and children with higher DMFT score (RRR: 1.04; 95% CI: 1.03; 1.06).

## 4. Discussion

This study addressed for the first time the magnitude and severity of malocclusion conditions in preadolescent Lebanese children, a comparison between public and private school children, and the association of social and behavioral factors with a wide range of malocclusion features.

### 4.1. Malocclusion

In addition to high malocclusion severity in all children, this study disclosed varying magnitudes of severity between the two school groups, depending on the malocclusion variable. The most prevalent variable was the overjet, which occurred in at least 20% of children. However, the statistically significant difference between OJ in PB (3.71 ± 1.77 mm) and PV (3.41 ± 1.70 mm) arguably may not be clinically significant.

**Figure 1:**
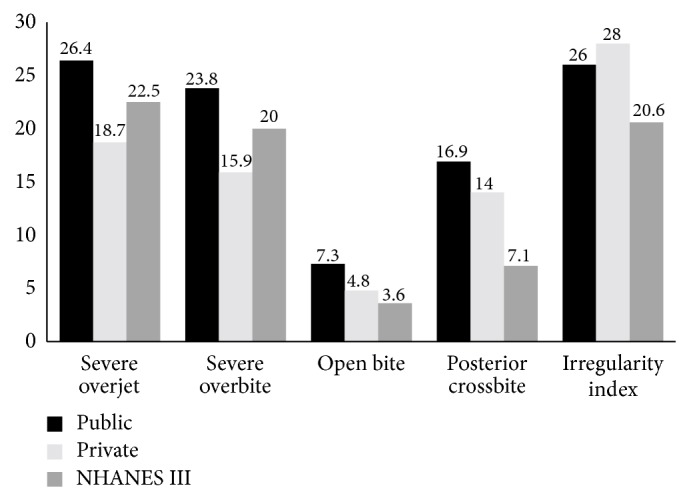
Percent distribution of students aged 8–11 by malocclusion characteristics and type of school (public and private) compared with the NHANES III findings.

For a more universal perspective, we compared our findings with the published data from the NHANES III survey, carried out between 1988 and 1999 on nearly 7000 individuals from different racial/ethnic and age groups [2]. Malocclusion in the NHANES was stratified on the overjet. Our findings regarding molar occlusion are consistent with other studies of Caucasian children [12, 13]. When malocclusion was classified on overjet, more similarities were found with the literature [2, 27] but the occlusion in public school children was most similar to the NHANES III data (Figure 1). Less Class II malocclusion and more Class I occlusions were found in the private schools (77.57% Class I, 16.96% Class II) compared to both NHANES III (74.8% Class I, 22.5% Class I) and public school (72.93% Class I, 23.69% Class II) data.

In some patients, the relationship between maxillary and mandibular molars falls between the three classes of malocclusion. Accordingly, the overjet was used as a more practical but not perfect proxy in various studies [27, 28]. In our sample, more than 9 of 10 subjects with an overjet greater than 6 mm had a Class II molar relationship, a finding consistent with other studies [28].

In other aspects of malocclusion, the following comparisons emerge (Figure 1).

- The prevalence of overjet and overbite in the moderate to severe range is greater in the NHANES III survey than in the PV schools, but less than in the PB schools. This disparity may relate to the wider range or lack of differentiation of socioeconomic backgrounds in the US survey compared to the differentiated socioeconomic levels of the PV and PB children in this study.
- A higher prevalence of open bite, posterior crossbite, and crowding in Lebanese school children compared to the NHANES III. The difference might relate to the higher prevalence/severity of sucking habits in our sample.
- A lower irregularity index in the NHANES III than in both groups. The higher incidence in males is similar to the NHANES III, while being inconsistent with other studies in which no differences [1] or higher female prevalence [29, 30] was found.

The epidemiology of malocclusion is significant because of multilevel impacts.

- Personal image: malocclusion may influence self-concept. Our findings on crowding (OR : 5.359) and crossbite (OR : 6.153) were reported by other investigators as risk factors for “global self-concept” (includes six domain-specific scales: social, competence, affect, academic, family, and physical) [31].
- Individual health: Our observations on anterior crossbite [underbite] (OR = 4.016) and molar relationship (OR = 1.661) match other findings that disclosed these characteristics as risk indicators for speech and chewing capabilities, respectively [32].
- Quality of life in general: patients with severe malocclusion scored poorer oral health-related quality of life (OHRQoL) than patients with less critical treatment need [33]. Also, orthodontic intervention would enhance some aspects of OHRQoL. More specific to the age bracket we investigated (6–11 years), early orthodontic intervention is beneficial because younger children have high self-esteem and body-image and expect orthodontics to improve their lives [33].

### 4.2. Associations

Associations are listed in 2 categories, those first found in this study and those corroborated in other studies.

#### 4.2.1. New/Different Findings

- Overbite was negatively associated with DMFT score. This finding might be partially explained by the association between mouth breathing (usually associated with open bite [34]) and increased risk of caries, through the reduction of the salivary flow that helps protect the teeth against decay [35].
- The multivariate analysis suggests that being in a private school is “protective” against increased overjet. The increased overjet might be linked to the environment in which the students live where some conditions (possibly higher rate of upper respiratory tract infections or pollution) enhance mouth breathing. Further research is needed to determine reasons for the differences.

#### 4.2.2. Findings Supported in the Literature

Consider the following:

- correspondence of family income and education [36],
- more prevalent sucking habits in PB children, possibly reflecting the reported increase in sucking habits of children feeling insecure, lonely, or stressed [37],
- higher proportion (3-fold) of mothers of PB children who smoked during pregnancy, concurring with prior conclusions that smoking during pregnancy is negatively correlated with educational level and socioeconomic status [38, 39],
- the association between overbite and sucking duration [40, 41], possibly explained by the link of sucking habits with tongue thrust and abnormal swallowing pattern [42],
- positive associations between posterior crossbite and age and between crossbite and the presence (and duration) of a sucking habit [43],
- the association between irregularity of incisors and DMFT [44, 45]. More caries may develop because of inappropriate brushing of the crowded incisors,
- the association between mouth breathing and increased irregularity score of the mandibular incisor [46]. Focused investigation is needed for definitive explanation.

Because all investigated children were past the primary dentition, comparisons are not possible with reports of association between sucking habits and increased overjet in this dentition [47, 48]. However, it is plausible that the higher prevalence of severe OJ in public school children was related to higher prevalence and intensity of sucking duration at an earlier age.

### 4.3. Implications for School Health

The findings underscore the need to reduce social disparities in oral health among Lebanese city pupils. The inequality stems from living conditions and unequal access to proper dental awareness/education and care. Short- and long-term strategies are considered to remedy this problem.

#### 4.3.1. Short-Term Recommendations

Regular annual dental screening is a public health service that should be generalized to all schools, not limited to mostly private and NGO-supported public schools, through the following:

- integrating orthodontic screening in the annual health evaluation, involving an orthodontist or trained dentist or dental hygienist, and basic screening tools. Several alternatives may be explored when funds are lacking: the assistance of civil NGO or charity organizations; volunteer dental examiners; coordination with orthodontic residency programs (possibly within a community service requirement for residency certification),
- the requirement by governmental health and educational agencies for all schools to institute an annual orthodontic/dental screening starting at age 7 years, the time recommended by the American Association of Orthodontists [24] or at least by the age of 8–8.5 years, the expected time for achieving the early mixed dentition,
- documentation by the pediatrician, during the child's regular medical screening, of mouth breathing and sucking habits, followed by referral for treatment of these habits. The prevalence/severity of certain aspects of malocclusion (posterior crossbite, irregularity index) would be decreased, oral health improved, awareness raised and potentially the screening protocol modified in pediatric residency.

#### 4.3.2. Long-Term Recommendations

Current dental insurance schemes are limited in Lebanon particularly for orthodontic treatment. Affordable access to orthodontic care is facilitated by the presence of 4 postgraduate orthodontic programs in the Beirut area. In other countries, oral healthcare is provided through private dental insurance and poorly to moderately funded public programs. In Europe, particularly Scandinavian countries characterized by their universal healthcare coverage, severe malocclusion is treated free of charge until a certain age (usually 18 years) and at low cost thereafter [49].

Such schemes might not be cost effective in Lebanon before studies and pilot programs help weigh the viability of any insurance plan before implementation. Focus on prevention may be more effective in the initial phases. Interceptive orthodontic treatment in US Medicaid patients has been effective in reducing malocclusion severity; some subjects might not require additional comprehensive orthodontic treatment at later stages [50, 51].

### 4.4. Research Considerations

A number of parents of children in private schools refused to enlist their children in the study because they had their own dentist. The lower percentage of private school respondents possibly impacted the rate and severity of malocclusion between the school groups. The study did not include children from the more expensive private schools, presumably representing higher living standards and different lifestyles, thus possibly altering some findings with potentially more divergence between both types of schools investigated.

Self-reporting might have affected the accuracy or underestimated some variables because of recall bias or misinterpretation of the question. Both limitations might have been diminished or overcome if the investigators had a direct interview with the parents.

Long-term follow-up on the screened subjects should confirm the reported associations and explore new ones among the studied parameters.

## 5. Conclusions

Malocclusion was more severe in preadolescent school children from lower socioeconomic background, indicating social disparities in oral health. Some associations were found between malocclusion and societal/behavioral parameters (e.g., sucking habits, prevalent in public school children, with open bite and posterior crossbite; crowding among mandibular incisors with DMFT score [indicative of oral hygiene]). Except for a lower prevalence of overjet/overbite in private schools, Lebanese school children have more severe malocclusion components than American children.

## Figures and Tables

**Table 1:** Sociodemographic variables.

Characteristics	School type		*P* value
	Public (*n* = 325)	Private (*n* = 330)	
Age (years)	8.49 ± 1.59	8.57 ± 1.31	NS

	%	%	
Gender			
Males	52.8	46.2	NS
Females	47.2	53.8	
Family income (LL)			
<500,000	33.6	1.4	0.000
500,000–999,999	49.4	14.2	
1,000,000–3,000,000	15.1	57.6	
>3,000,000	2.1	26.4	
Education of informant			
Low (illiterate-primary-elementary)	45.4	7.7	0.000
Average (secondary-intermediate)	44.1	20.0	
High (college/university)	10.5	72.4	

**Table 2:** Health and behavioral characteristics of child and mother.

Characteristics	School type		*P* value
	Public (*n* = 325) %	Private (*n* = 330) %	
Chronic diseases			
Yes	13.4	9.1	NS
No	86.6	90.9	
Mouth breathing			
Yes	9.8	7.7	NS
No	90.2	92.3	
Sucking habits			
Yes	19.56	14.9	0.030
No	80.43	85.1	
Maternal smoking during pregnancy (cigarettes)	20.4	7.0	0.000
Feeding method			
Breast	53.3	31.0	NS
Bottle	22.7	24.2	
Both	24.0	44.8	

**Table 3:** Percentage distribution of malocclusion characteristics in public and private school children.

Measures	School type		*P* value^*^
	Public (*n* = 325)	Private (*n* = 330)	
Overjet (%)			
1-2 [ideal]	27.4	36.3	0.022
3-4 [mild]	46.2	45.0	
>4 [mod-sev]	26.4	18.7	
Mean OJ (mm)	3.71 ± 1.77	3.41 ± 1.7	0.032
Anterior crossbite (%)			
0 [mild]	5.23	0.9	0.008
−1 to −2 [moderate]	5.5	6.6	
−3 to −4 [severe]	0.3	0.9	
<−4 [extreme]	0.0	0.0	
Occlusion^**^ (%)			
I	72.93	77.57	0.002
II	23.69	16.96	
III	3.38	5.45	
Midline diastema (>2 mm)	16.1	10.5	0.036

^*^Chi-square.

^**^Cl I, Cl II, and Cl III classified based on OJ (ideal: 1–4; >4: Cl II; reverse overjet: Cl III).

**Table 4:** Associations in percentage between components of malocclusion and other variables.

Associations				*P* value
Overjet	1-2 [ideal]	3-4 [mild]	4< [mod-sev]	

Age				
(6-7)	38.6	41.9	19.5	0.033
(8–11)	28.2	48.0	23.8	
DMFT	4.79 ± 3.98	5.36 ± 4.24	6.18 ± 4.12	0.030

Overbite	0–2 [ideal]	3-4 [moderate]	5< [mod-sev]	

Age				
(6-7)	63.9	24.4	11.8	0.033
(8–11)	48.7	27.4	23.8	
Plaque index (PI)	1.27 ± 0.21	1.24 ± 0.16	1.30 ± 0.22	0.049

Posterior crossbite	Present	Not present		

Sucking habits				
Present	75.8	24.2		0.005
Not present	86.9	13.1	

**Table 5:** Multivariate analysis of associations between categories of malocclusion and other variables.

Overjet^*^				
Variable	RRR	Robust SE	95% CI	*P* value
Mild				
Age (6-7 versus 8–11)	1.35	0.16	[1.067; 1.71]	0.013
Moderate to severe				
Plaque index	0.93	0.024	[0.888; 0.983]	0.009

Overjet (continuous measurement)				
Variable	*β*	Semi-Robust SE	95% CI	*P* value

Age	1.15	0.051	[1.04; 1.28]	0.004
School type	0.9	0.04	[0.831; 0.974]	0.009
Family income^**^				
500,000–999,999	1.229	0.070	[1.07; 1.41]	0.003
1,000,000–3,000,000	1.328	0.044	[1.209; 1.447]	0.000
>3,000,000	1.205	0.123	[0.94; 1.535]	0.131

Overbite^*^				
Variable	RRR	Robust SE	95% CI	*P* value

Mild				
Age (6-7 versus 8–11)	1.709	0.294	[1.21; 2.39]	0.002
Moderate to severe				
Age (6-7 versus 8–11)	2.238	0.880	[1.035; 4.83]	0.040
Sucking duration	0.983	0.004	[0.97; 0.992]	0.000
DMFT	0.930	0.033	[0.866; 0.999]	0.048

Posterior crossbite				
Variable	OR	Semi-Robust SE	95% CI	*P* value

Age (6-7 versus 8–11)	1.29	0.041	[1.188; 1.39]	0.000
Sucking duration	1.014	0.001	[1.011; 1.185]	0.000

Irregularity index^*^				
Variable	RRR	Robust SE	95% CI	*P* value

Mild				
Mouth breathing	2.612	0.362	[1.99; 3.428]	0.000
Moderate to severe				
Gender	1.692	0.189	[1.359; 2.1]	0.000
DMFT	1.049	0.0082	[1.033; 1.065]	0.000

^*^Ideal category as base outcome.

^**^ <500.000 as base outcome.

*β*—slope of regression; OR: odds ratio; RRR: relative risk ratio.
